# Stress and the Emerging Roles of Chromatin Remodeling in Signal Integration and Stable Transmission of Reversible Phenotypes

**DOI:** 10.3389/fnbeh.2017.00041

**Published:** 2017-03-15

**Authors:** Ian C. G. Weaver, Austin C. Korgan, Kristen Lee, Ryan V. Wheeler, Amos S. Hundert, Donna Goguen

**Affiliations:** Department of Psychology and Neuroscience, and Department of Psychiatry, Dalhousie UniversityHalifax, NS, Canada

**Keywords:** brain development, epigenome, chromatin remodeling, DNA methylation, histone modification, transgenerational inheritance

## Abstract

The influence of early life experience and degree of parental-infant attachment on emotional development in children and adolescents has been comprehensively studied. Structural and mechanistic insight into the biological foundation and maintenance of mammalian defensive systems (metabolic, immune, nervous and behavioral) is slowly advancing through the emerging field of developmental molecular (epi)genetics. Initial evidence revealed that differential nurture early in life generates stable differences in offspring hypothalamic-pituitary-adrenal (HPA) regulation, in part, through chromatin remodeling and changes in DNA methylation of specific genes expressed in the brain, revealing physical, biochemical and molecular paths for the epidemiological concept of gene-environment interactions. Herein, a primary molecular mechanism underpinning the early developmental programming and lifelong maintenance of defensive (emotional) responses in the offspring is the alteration of chromatin domains of specific genomic regions from a condensed state (heterochromatin) to a transcriptionally accessible state (euchromatin). Conversely, DNA methylation promotes the formation of heterochromatin, which is essential for gene silencing, genomic integrity and chromosome segregation. Therefore, inter-individual differences in chromatin modifications and DNA methylation marks hold great potential for assessing the impact of both early life experience and effectiveness of intervention programs—from guided psychosocial strategies focused on changing behavior to pharmacological treatments that target chromatin remodeling and DNA methylation enzymes to dietary approaches that alter cellular pools of metabolic intermediates and methyl donors to affect nutrient bioavailability and metabolism. In this review article, we discuss the potential molecular mechanism(s) of gene regulation associated with chromatin modeling and programming of endocrine (e.g., HPA and metabolic or cardiovascular) and behavioral (e.g., fearfulness, vigilance) responses to stress, including alterations in DNA methylation and the role of DNA repair machinery. From parental history (e.g., drugs, housing, illness, nutrition, socialization) to maternal-offspring exchanges of nutrition, microbiota, antibodies and stimulation, the nature of nurture provides not only mechanistic insight into how experiences propagate from external to internal variables, but also identifies a composite therapeutic target, chromatin modeling, for gestational/prenatal stress, adolescent anxiety/depression and adult-onset neuropsychiatric disease.

## Introduction

Brain development and the emergence of antecedent fear and anxiety-like behaviors that increase the risk for severe mood and psychotic disorders—i.e., major depressive disorder (MDD), schizophrenia (SCZ) and bipolar disorder (BPD)—are influenced by inherited and non-inherited (i.e., acquired during life) factors, where environmental experience contributes to disease onset (Pine and Fox, 2015). Major psychiatric disease risk is largely attributed to germline mutations, with heritability estimates ranging from 30% to 80% (Cardno et al., 1999; Kendler and Prescott, 1999). The discordance between monozygotic (identical) twins demonstrates that non-inherited factors, such as environmental perturbations or somatic mutations, may also drive disease etiology (Petronis, 2010). The latency between exposure and disease emergence suggests that the environment propagates stable changes that have the potential to manifest later in life. Identification and characterization of candidate genes and mechanism(s) by which early experiences direct key cell neurodevelopmental pathways are essential to establish appropriate interventions that protect child neurodevelopment—i.e., the dynamic inter-relationship between genetic, brain, cognitive, emotional and behavioral processes across the developmental lifespan.

Inter-individual variations in physical, cognitive and socioemotional growth have been traditionally examined under the conceptual framework of gene–environment (G × E) interactions (Dick et al., 2010; Gershon et al., 2011). Herein, animal studies have identified genetic sequences that influence behavior, and human genome-wide association studies (GWAS) have linked specific genotypic variants—aneuploidy, copy number variants (CNVs), indels, retrotransposition and single nucleotide polymorphisms (SNPs)—to certain personality traits, including psychiatric disorders (Malhotra and Sebat, 2012; Purcell et al., 2014) and anxiety (Murakami et al., 1999; Binder et al., 2004). However, such relations are unable to explain the vast majority of the inter-individual variation in the population (Dick et al., 2010; Gershon et al., 2011). Cellular variables and alternate molecular mechanisms have since been examined, including epigenetic programming. Waddington (1942) introduced the term epigenetics to describe the mechanisms that are involved in programming identical genes differently in different organs during embryogenesis. Although often debated (Henikoff and Greally, 2016), epigenetics represent mitotic or meiotic heritable patterns of DNA methylation and chromatin protein modifications that affect how DNA is packaged, and the stable transmission of gene expression programs and phenotypes (Wolffe and Matzke, 1999). Microarray-based and next generation sequencing platforms have led to methods which provide high-resolution genome-wide distribution of epigenetic (collectively called epigenomic) modifications in normal and diseased states.

Depending on the genomic location, a chromatin modification could have a range of effects on cellular function by altering gene expression or transcript splicing, to further modify chromatin or to reverse an existing chromatin modification. Considering the highly-networked state of the brain, a small number of chromatin modifications that affect cellular function could have far-reaching effects on neuronal circuitry and behavioral traits. The nature and degree to which molecules—that either attach (“writers”) or erase (“erasers”) modifications to chromatin or that bind (“readers”) to a specific modified site—may contribute to antecedents and emergence of neuropsychiatric disorders in humans is currently under investigation (Singh et al., 2016). A long-standing question has been to determine the mechanistic link between early environmental experiences and permanent alterations in the brain and their contribution to disease risk and progression later in life. Here, we discuss the major molecular (epi)genetic mechanisms that control stable gene expression (temporal and spatial) in the brain and generated in response to physiological or pathological signals, the environment, challenges associated with studying the contribution of these molecular (epi)genetic changes to complex behavioral phenotypes, and future directions for understanding the manifestation of stress in humans.

## Dynamic Organization of Chromatin Structure and Function

**Figure 1:**
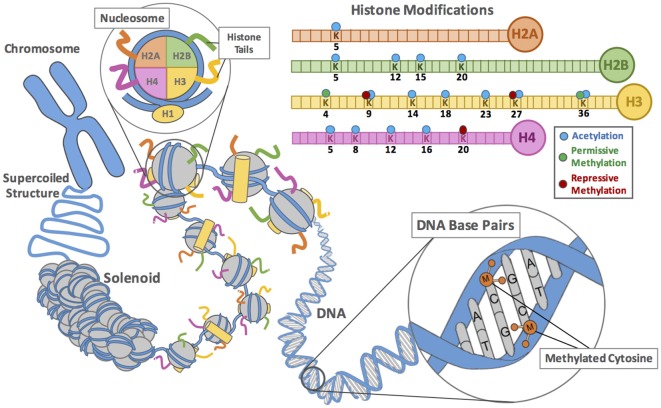
**The dynamic (epi)genome: DNA methylation, histone post-translational modifications and chromatin structural organization.** Within the nucleus of eukaryotic cells, chromosomes are composed of DNA coiled around an octamer of histone proteins to form nucleosomes, the basic repeating unit of chromatin. Histone H1 proteins stabilize the coupling, wrapping and stacking of nucleosomes into a 30 nm solenoid and higher order supercoiled chromatin fiber. The histone octamers are composed of four pairs of histone (H2A, H2B, H3, and H4) proteins, which have globular domains and N-termini tails that protrude from the nucleosome (H2A also has a C-terminal tail). Each histone tail can undergo numerous post-translational modifications. The most common forms of mammalian acetylation and methylation modification of lysine (K) residues are shown. Additionally, mammalian DNA can be chemically modified by methylation and hydoxymethylation (M) of the five position of the cytosine base of 5′-cytosine-phosphodiester-guanine (CpG) dinucleotides. Chromatin structure directs the activity (expression) of genes: genes within tightly packed nucleosomes are silenced, whereas genes within relatively spaced nucleosomes are actively transcribed (expressed). The process of remodeling chromatin into domains of different transcriptional potentials is regulated by reciprocal changes of DNA methylation and histone modification in response to in response to extrinsic cues and/or changes in intrinsic properties of cells (see text for details).

Cells in multicellular organisms are structurally and functionally heterogeneous due to differential gene expression, which is controlled by dynamic organization of the genome into chromosome territories and domains of different transcriptional potential (Allis and Jenuwein, 2016). In the nucleus of a eukaryotic cell, gene expression is primarily controlled by chromatin structure (Figure 1). The nucleosome is the fundamental unit of chromatin consisting of ~147 base pairs of DNA wrapped tightly around an octamer of histone proteins (composed of two H2A-H2B dimers and a H3-H4 tetramer), termed the nucleosome core. During nucleosome formation, two H3-H4 dimers are first assembled on DNA, where they form a subnucleosomal structure called the tetrasome. Two H2A-H2B dimers are then incorporated into the tetrasome, to form the mature nucleosome. Histone linker protein H1 associates with the DNA between nucleosome cores (linker DNA) and functions in the compaction of chromatin into higher-order structures that comprise chromosomes (Figure 1). The formation of specific nucleosome arrays along the genome (referred to as the “beads on a string”) confers different structural and functional chromatin states—for example, promoter regions of actively transcribed genes are depleted of nucleosomes, with nucleosome occupancy progressively increasing into coding regions. Strong DNA-histone association results from binding of the negatively charged DNA phosphate backbone with the many positively charged (basic) amino acids (e.g., lysine, arginine) of the nucleosomal histones (Figure 1). Genes associated with this tightly compacted form of DNA, termed heterochromatin, are transcriptionally silent. Heterochromatin is thus an assembly that is inhibitory to cellular processes requiring direct interactions with the genome. Alteration of chromatin structure, termed “chromatin remodeling”, and DNA accessibility from a closed to an active (euchromatin) state, can be accomplished by: (i) adenosine triphosphate (ATP)-dependent complexes, which modulate histone-DNA association; and (ii) covalent modification of the core nucleosomal histones, which mediate the transcriptional activity (Ronan et al., 2013). This molecular mechanism is regulated by a variety of chromatin modifications, including DNA methylation, non-coding RNAs (micro RNAs, long non-coding RNAs and others), polycomb-group proteins and post-translational histone modifications.

This dynamic organization of chromatin structure prevents chromosome breakage and allows control of gene expression as well as replication and distribution of DNA in mitotic and meiotic cell division, thereby regulating patterns of gene expression and maintenance of cellular phenotype across generations. However, the extent to which chromatin organization is passed on through cell division during development depends heavily on cell type. Chromatin modifications that occur in early progenitor cells during embryogenesis are passed on to most cells of the brain and body. Alternatively, chromatin modifications that occur in neural progenitors in the neurogenic niche of the adult brain are transmitted to a few number of cells. Beyond cell identity, epigenetic mechanisms in single neurons can be modified in response to a variety of intrinsic (e.g., transcription factors, retro-transposition, prion-protein-like mechanisms) and external stimuli (e.g., nutrition, toxicants, drug exposure) to provide experiential identity through persistent changes in gene expression, cell function and phenotype, and might even be inherited transgenerationally (Bailey et al., 2004; Ballas and Mandel, 2005; Muotri and Gage, 2006). These emerging concepts have biological relevance not only to cell/tissue homeostasis during normal development and ageing, but also in the onset of physical and mental illness.

## Regulation of Chromatin by Histone Modifications

The dynamic structure of chromatin is dependent on the histone tails of the core histones, which interact with nucleosomal and linker DNA and therefore not only play critical roles in gene regulation but also in the formation of higher-order chromatin (Figure 1). Notably, the N-terminal tails of H2B, H3 and H4 and C-terminal tail of H2A are accessible to histone modifying enzymes in the nucleus and can undergo post-translational modifications, including: lysine acetylation, lysine mono-, di-, or tri-methylation and arginine mono- or di- (asymmetric or symmetric) methylation, serine/threonine phosphorylation, lysine biotinylation and butyrilation, arginine citrulation, but also mono-ubiquitination, SUMOylation, poly-ADP-ribosylation, deamination, carbonylation and proline isomerization, along with their reversed processes (Zhao and Garcia, 2015). Additionally, the chromatin fiber can be modified by the substitution of canonical histones for histone variants—a process termed nucleosome subunit exchange. Together with post-translational modifications, histone variants alter the physical properties of nucleosomes to provide chemical signposts that serve to recruit specific nuclear proteins through recognition of the particular modification and non-allelic histone isoform (for H2A, H2B and H3) involved, as well as the context and surrounding histone modifications. For example, the H2A variant H2A.X regulates chromatin structure and gene expression, while the phosphorylated form (γH2A.X) helps recruit DNA repair proteins in response to severe DNA damage—as a result of environmental insult, metabolic mistake, or programmed process (Suberbielle et al., 2013). Exchange of histone H2A for variant H2A.Z affects nucleosome stability and is involved in transcriptional control, chromosome segregation and gene silencing (Marques et al., 2010). Recent studies suggest H2A.Z is also necessary for acetylation and ubiquitination of histones and promotes remodeling of the local chromatin structure to enable the DNA repair machinery to access sites of DNA damage (Xu et al., 2012). Thus, each histone modification can affect chromatin fiber stability and the capacity to attract protein complexes that either compact the chromatin even further, or facilitate accessibility of transcription machinery (Figure 1). The effects of histone modification on gene activity (expression) depends on the identity, location and extent of each modification that regulates downstream processes, such as gene transcription, DNA repair, DNA replication and programed cell death (apoptosis). Translating the “histone code” and understanding how histone modifications are regulated could lead to therapies that shut down or turn on genes in diseases that have aberrant patterns of gene expression. Consequently, chromatin marks and histone modifying enzymes have emerged not only as promising biomarkers for disease diagnosis and prognosis, but also informative for distinguishing disease subtypes and identifying suitable treatments (Bock et al., 2016; Libertini et al., 2016a,b; Rendeiro et al., 2016).

In general, histone deacetylation, biotinylation and SUMOylation repress gene transcription, while histone acetylation and phosphorylation act as activators of gene expression. Depending on the histone residue being modified, methylation and ubiquitination can either repress or activate gene transcription. In order to define a precise functional chromatin environment, histone modifications often demonstrate interdependence—for example, histone acetylation, phosphorylation and ubiquitination can all be regulated by histone methylation (for review see Izzo and Schneider, 2010). Beyond this, trans-nucleosome cross-talk between post-translation modifications and histone modifying enzymes contribute to the establishment and maintenance of chromatin domains with different transcriptional potentials. Histone modification enzymes themselves are not sequence specific and have to be recruited to particular genomic regions by interactions with transcription factors. Some transcription factors (e.g., c-Jun, GATA1, NRF1) are uniquely associated with euchromatin, while others (e.g., ZFN274, KAP1, SETDB1) are only associated with heterochromatin (Ernst et al., 2011; Thurman et al., 2012). The relation between transcription factor function and chromatin state creates a mechanistic connection through which developmental and environmental cues might alter chromatin domains and the expression of specific genes in post-mitotic neurons. The best characterized histone modifications are histone acetylation/deacetylation and histone methylation/demethylation, and the enzymes and molecular mechanisms governing these marks are discussed below.

## Histone Acetylation—Deacetylation

In the 1960s, Allfrey proposed that histone acetylation was associated with transcriptional potential (Allfrey and Mirsky, 1964; Allfrey et al., 1964). Subsequent studies have helped establish the causal relationship between histone modifying enzymes, histone marks and gene regulation (Figure 1). Acetylation at lysine (K) residues on the amino-terminal tails of histone proteins (e.g., H3K9, H3K14, H3K36, H4K8, H4K16) is mediated by both histone acetyltransferases (HATs) and histone deacetylases (HDACs), which are recruited by activator and corepressor proteins, respectively (Kalkhoven, 2004; Wang et al., 2009). For example, the addition of an acetyl group (acetylation) on lysine 9 of histone H3 (H3K9Ac) in gene promoter or enhancer regions by HAT enzymes (e.g., nuclear type A proteins, GCN5, p300/CBP and TAFII250) is associated with transcriptionally active euchromatin. Alternatively, removal of the acetyl group (deacetylation) by HDAC enzymes (e.g., nuclear class 1 proteins, HDAC 1, 2, 3 and 8) is associated with transcriptionally inactive heterochromatin (Grunstein, 1997; D’Alessio et al., 2007).

Histone acetylation is a process that is dependent on the enzyme ATP-citrate lyase, which converts glucose-derived citrate into acetyl-coenzyme A (acetyl-CoA; Takahashi et al., 2006). HATs transfer an acetyl group from acetyl-CoA to form ε-N-acetyllysine. Acetyl-CoA is produced by glycolysis and other catabolic pathways such as the β-oxidation of fatty acids, and plays a key regulatory role as a substrate for the citric acid cycle and as a precursor in the synthesis of fatty acids and steroids. Mitochondrial and nucleocytosolic acetyl-CoA pools are therefore a rate limiting step, coupling metabolism with chromatin remodeling and endocrine function (Wallace and Fan, 2010). In turn, mitochondrial dynamics permit reversible modulation of gene expression, growth and reproduction potential in response to changes in energy demand and nutrient supply (Wallace, 2010)—i.e., for bioenergetic adaptation to metabolic demands—and may affect a myriad of cellular and biochemical processes in which acetylation and metabolism intersect, such as aging and neurodegenerative disease states (e.g., Alzheimer’s disease (AD), Parkinson’s disease (PD); Liesa and Shirihai, 2013). HATs are broadly classified into two different classes, based on their functional localization: (1) type A HATs, located in the nucleus and contain a bromodomain, and (2) cytoplasmic type B HATs, that modify newly synthesized histones before their assembly into nucleosomes (Sterner and Berger, 2000; Roth et al., 2001). On the other hand, HDACs are divided into five main classes based on their sequence homology and expression patterns. Class I, IIA, IIB and IV HDACs are Zn-dependent deacetylases, whereas the Class III HDACs are nicotinamide adenine dinucleotide (NAD)-dependent deacetylases (Sterner and Berger, 2000; Roth et al., 2001).

Through animal models, HDAC inhibitors (HDACis) have been recognized as potentially useful therapeutic interventions for the cognitive impairments associated with chronic stress, neurodevelopmental disorders and neurodegeneration (reviewed in Dietz and Casaccia, 2010; Didonna and Opal, 2015). These inhibitors include hydroxamates, such as vorinostat (SAHA) and trichostatin A (TSA); short chain fatty acids, such as sodium butyrate (SB) and valproic acid (VPA); cyclic tetrapeptides, such as apicidin or depsipeptide, amides, benzamides, epoxides, ketones and lactones. Although VPA has long been used as an anticonvulsant drug for the treatment of migraines, as well as partial and generalized seizure disorders in individuals with epilepsy and BPD, the further development of similar pan HDACis to treat neurobiology and neurological diseases has been hampered by off-target as well as severe side effects (Dinarello et al., 2011; Soragni et al., 2011). Similar to therapeutic strategies to enhance the anticancer efficacy of HDAC inhibitors, isoform-selective and/or class-selective HDAC inhibition in combination with other epigenetic modulators and/or other chemotherapeutic agents may not only reduce these off-target effects, but also provide a potential strategy to respond to resistance to current therapies in the treatment of human neuropathology.

## Histone Methylation—Demethylation

Methylation of lysine or arginine residues on amino-terminal tails of histone proteins (Figure 1) is controlled by the activity of both histone lysine or arginine methyltransferases (HTMs; e.g., EZH2, G9a, MLL, Suv39H1/2) and histone lysine or arginine demethylases (HDMs; e.g., JARID1d, Utx; Greer and Shi, 2012). Lysine residues can carry either mono-, di- or trimethyl moieties on their amine group (e.g., H3K4, H3K9, H3K27, H3K36, H3K79 and H4K20). Alternatively, arginine residues can house mono- or di-methyl (me2) moieties on their guanidinyl group, in either symmetric (me2s) or asymmetric (me2a) configuration. Methylation of histone H3 on lysines 4, 36, 79 (H3K4, H3K36 and H3K79) is generally associated with poised or active gene transcription, whereas methylation of histone H3 on lysine 9, 20, 27 (H3K9, H3K20 and H3K27) are hallmarks of silenced or heterochromatic regions (Ohm and Baylin, 2007). H4K20 modification is also involved in recruiting the checkpoint protein CRB2 to sites of DNA damage, suggesting that HTMs may have many different roles in the cell. Methylation of histone H3 on arginines 2, 8, 17 and 26 (H3R2, H3R8, H3R17 and H3R26) and H4 on arginine 3 (H4R3) can be either activatory or repressive for transcription. The distribution, recognition and regulation of histone lysine/arginine methylation is of major interest given their role in the regulation of chromatin and gene expression and importance in multiple pathways in development and disease, including metabolic and neurological disorders (Landgrave-Gómez et al., 2015).

The majority of HMTs have a conserved SET (Suppressor of variegation, Enhancer of Zeste, Trithorax) catalytic domain and their activity towards the lysine and arginine residues can result in mono-, di-, or tri methylation state of the amino acids. Understanding the specificity of different SET-domain enzymes and which human diseases can arise from changes in HMT–binding (reading) domains may provide novel targets for therapeutic drugs. Methylation of lysine and arginine residues on the amino-terminal tails of histone proteins is dependent on the methyl donor *S*-adenosyl methionine (SAM), SAM itself is derived in part from dietary methyl group intake—e.g., choline, methionine, or methyl-tetrahydrofolate—further linking metabolism with chromatin remodeling and cellular physiology.

Histone demethylases, on the other hand, can be classified into two classes: (1) Lysine Demethylase-1 (KDM1) family, also known as LSD1, are nuclear flavin adenine dinucleotide (FAD)-dependent amine oxidases; and (2) the Jumonji C (JmjC) domain containing demethylases (JHDMs), which are Fe(II) and α-ketoglutarate-dependent dioxygenase enzymes (Fodor et al., 2006; Tsukada et al., 2006; Whetstine et al., 2006). Unlike LSD1 enzymes, the JmjC class of enzymes are able to demethylate trimethyl- lysine histones (Klose et al., 2006). Given that LSD1 and JmjC histone demethlyases both require oxygen to function, the status of histone methylation is influenced by oxygen concentration. Cells and/or tissues become hypoxic when the demand for cellular growth and metabolism surpasses that of the oxygen supply. Hypoxia is an important factor in the pathology of a number of human diseases, including cancer, diabetes, ageing and stroke/ischemia. Initial results of current clinical trials with inhibitors of various lysine methyltransferases (e.g., DOT1L and EZH2) and demethylases (e.g., LSD1) for cancer therapy will likely guide the future clinical development for new histone methylation modifiers and different therapeutic markers (Morera et al., 2016).

In summary, histone modification enzymes require common metabolic intermediates (e.g., acetyl CoA, ATP, biotin, FAD/NAD^+^ and SAM) and their intranuclear levels are dependent on the metabolic state of the cell. Changes in local concentrations of key cellular metabolites can affect enzyme functions, acting as substrates or inhibitors of covalent modification to histone tails (Figure 1). Furthermore, levels and turnover of metabolic intermediates are influenced by dietary and nutrient intake, metabolic status (hypoxia, hyperglycemia, redox status, inflammation, oxidative stress), as well as endocrine unbalance and disease that, in turn, can alter histone modification enzyme expression levels.

## Regulation of Chromatin by DNA Methylation—Demethylation

DNA methylation is the primary epigenetic mark (often considered the “fifth base”) that regulates the formation of heterochromatic regions in the genome, with crucial roles in control of gene expression in both physiological and pathological conditions. A large proportion of the neuronal genome is under cytosine methylation regulation (Figure 1). In mammals, DNA methylation is predominantly found at cytosine residues in CpG-3′ dinucleotides to form 5-methylcytosine (5mC; Bestor, 1990; Jaenisch and Bird, 2003). The majority (~75%) of CpG dinucleotides are methylated. Genomic regions of enriched CpG content (CpG islands) are associated with ~60% of human gene promoters and predominantly hypomethylated. Early in development, however, ~4% of these gene promoter regions become methylated and transcriptionally silenced in a tissue-specific pattern (Borgel et al., 2010). Conversely, gene body DNA methylation is coupled to transcriptional activation, as well as translation elongation efficiency and protein-production rate (Lister et al., 2009). Silencing of repetitive elements within the mammalian genome (e.g., LINEs and SINEs including Alu elements in humans) by DNA methylation prevents aberrant expression that could cause chromosomal instability, translocations and gene disruption due to transposition events (Muotri and Gage, 2006). DNA methylation is also involved in the silencing of autosomal genes in a parent-of-origin manner, termed imprinting (Kelsey, 2011). Herein, the methylation status of the regulatory elements controlling genomic imprinting (e.g., imprinting control regions (ICRs)) dictates whether the paternal or maternal allele is expressed. The “imprintome” refers to the genomic repertoire of these differentially methylated regions, rather than the genes they regulate (Jirtle, 2009). A similar gene-dosage reduction is seen in X chromosome inactivation in females. DNA methylation is further linked to nuclear organization, concentrating in dense silenced heterochromatin regions. Allele-specific DNA methylation (ASM) reflects tissue-specific *cis*-regulatory influences of DNA polymorphisms on epigenetic status (Tycko, 2010), whereas compromised DNMT1 function at CpG sites (Chen et al., 1998) and deposition of methyl groups at certain CpG sites (Pfeifer, 2006) have been shown to enhance genetic variation leading to changes in gene expression and cell function (Chen et al., 1998), suggesting that differentially methylated CpG sites serve as evolutionarily established mediators between the genetic code and phenotypic diversity.

The DNA methyltransferases (DNMTs)—DNMT1, DNMT2, DNMT3A, DNMT3B and DNMT3L—are a family of enzymes that write the patterns of DNA methylation (Okano et al., 1999). These enzymes are all expressed in the central nervous system (CNS) and are dynamically regulated during development (Goto et al., 1994; Feng et al., 2005). The recognition and selective binding to hemi-methylated DNA by maintenance methyltransferase, DNMT1, ensures methylation patterns are faithfully copied from parental to daughter strand during DNA replication (Bestor, 1988, 1992). Methylation within critical regulatory regions of genes (transcription factor binding sites and enhancer elements) can silence gene expression by either directly blocking access and binding of transcription factors (Watt and Molloy, 1988; Tate and Bird, 1993), or through recruitment of methyl-CpG binding domain (MBD) proteins such as MeCP2 and MBD proteins 1–4 that bind to the methylated DNA and recruit co-proteins such as SIN3A and histone modification enzymes, leading to heterochromatin formation (Helbo et al., 2017). Non-CpG cytosine methylation (i.e., mCpH, where H = adenine (A), cytosine (C) or thymine (T)) constitutes ~25% of the DNA methylome and has also been linked to early neural development (Lister et al., 2013) and adult mammalian brain function (Guo et al., 2014). Neuronal CpH methylation is enriched in regions of low CpG density and, similar to CpG methylation, is depleted at protein-DNA interaction sites and functionally can repress transcription in neurons. As CpG dinucleotides form only ~1% of the mammalian genome, CpH methylation may therefore function to increase the local density of methylated cytosine in neurons in the absence of additional CpG dinucleotide methylation. Other modifications of cytosine in DNA include 5-carboxyl-cytosine and 5-hydroxymethylcytosine (5-hmC) formation, which forms ~40% of modified cytosines in neurons, increases in the brain with age and in response to neuronal activity, including acute stress (Song et al., 2011; Szulwach et al., 2011).

During mammalian development, DNA methylation marks are globally removed from both the maternal and paternal genomes at fertilization to ensure totipotency (Reik and Surani, 2015). Specific methylation patterns are re-established by the *de novo* methyltransferases DNMT3A and -3B, and modulated by DNMT3L (Okano et al., 1999). DNA methylation has long been considered a stable, static modification with few mechanisms for removal of the methyl group; leading to studies suggesting passive (DNA replication-dependent; Morgan et al., 2005) vs. active (enzymatically driven, DNA replication independent; Bhattacharya et al., 1999; Brown et al., 2008) processes. The rediscovery of 5hmC (Kriaucionis and Heintz, 2009; Tahiliani et al., 2009) led to the identification of a family of enzymes known as ten-eleven translocation 1–3 (TET1–3) with the ability to convert 5mC to 5hmC in an oxidation- driven reaction that generates other intermediates (that is, 5-formylcytosine (5-fC) and 5-carboxylcytosine (5-caC); Tahiliani et al., 2009; Ito et al., 2010). Enzymatic excision of 5hmC by DNA glycosylases (termed base excision repair) may follow, replacing 5-hmC with cytosine resulting in active DNA demethylation and transcriptional activation (He et al., 2011).

Aberrant DNA methylation patterns and expression and/or activities of DNMTs are involved in several pathologies, from cancer to neurodegeneration (Zwergel et al., 2016). In cancer cells, anti-proliferation/tumor suppressor genes are frequently silenced by promoter CpG methylation, which led to the pursuit of DNMT inhibitors (DNMTi) as potential cancer therapeutics to reactivate these genes and stop or even reverse tumor growth and cell invasiveness. These inhibitors include nucleoside analogs, such as 5-azacytidine (Azacitidine), and more stable and less toxic 5-aza-2-deoxycytidine (decitabine), 5-fluoro-2-deoxycytidine (FdCyd), SGI-110 and Zebularine that intercalate into DNA during replication and inhibit DNMT1 activity; as well as other small molecule inhibitors that are not incorporated into DNA—such as RG108 (N-Phthalyl-1-tryptophan) that binds to the catalytic site of DNMTs causing inhibition of DNA methylation (Brueckner et al., 2005; Zheng et al., 2008) and the antisense oligonucleotide MG98 (2′-*O*-CH_3_-substituted phosphorothioate oligo deoxynucleotide) that targets the 3′ UTR of DNMT1, blocks translation of the *Dnmt1* mRNA, thereby causing a decrease in DNA methylation (Stewart et al., 2003; Klisovic et al., 2008). DNMTi treatment can also lead to widespread gene-body demethylation and transcriptional downregulation of overexpressed oncogenes, suggesting convergent mechanisms for DNMTi mediated cell growth inhibition (Yang et al., 2014). For example, key molecular targets and DNA methylation marks linked to hormone-receptor-targeted therapy inhibition in triple-negative breast cancer (Coyle et al., 2016) provide further insight for novel therapeutic intervention strategies for cancer pathology. Similar to histone-modifying enzymes, several natural compounds such as polyphenols, flavonoids and antraquinones (e.g., (-)-epigallocatechin-3-gallate and laccaic acid A) inhibit DNMT activity and/or expression, resulting in the re-expression of anti-proliferation/tumor suppressor genes, tumor growth inhibition and cell death (Lee et al., 2006a). However, these non-nucleoside analog inhibitors are less potent than the nucleoside analogs and require further optimization (Chuang et al., 2005).

Neurodegenerative disorders (including, AD, dementia with Lewy bodies, PD, Down’s syndrome) share similar aberrant CpG methylation profiles at DMRS that overlapped gene promoter regions of common genes involved in a variety of cellular signaling pathways (e.g., ErbB, TGFβ, Wnt, MAPK, Neurotrophin, p53) that influence brain development and function (Sanchez-Mut et al., 2016). These findings suggest not only that different neurodegenerative diseases emerge from similar pathogenetic mechanisms, but also that DNA methylation is key in the aberrant changes in gene expression associated with cell survival. When administered directly into the brain tissue of rodents, DNMTi treatment blocks neurotoxicity associated with Huntington disease (Pan et al., 2016), while haploinsufficiency of Dnmt1 protects against irreversible damage following acute ischemia and recurring stroke (Endres et al., 2000, 2001), suggesting that DNA methylation-targeted drugs may rescue CNS functions after injury, promote neuron survival and prevent progressive dementia. However, DNMTi treatment can also disrupt synaptic plasticity and impair hippocampal learning and memory, and modulate reward and addiction behaviors (Sen, 2015). Furthermore, overexpression of the TET1 protein (which promotes 5hmC formation and active demethylation) results in increased expression of memory-associated genes in neurons as well as contextual fear memory impairment (Kaas et al., 2013). Understanding the complexity of DNA methylation (and histone modification) and the ability to epigenetically reprogram gene expression in differentiated cells, such as neurons, is therefore of major importance to cognitive research examining not only the role of emotions in information processing but also the effects of dysregulation on decision-making, including emotional states in social withdrawal, impulsivity, substance dependence in neuropsychiatric disorders and age-related neurodegenerative diseases.

## Inter-Relations Between Histone Modification and DNA Methylation and Transcriptional States

As described above, gene expression requires the alteration of chromatin domains from condensed heterochromatin to a transcriptionally accessible euchromatin and DNA demethylation. Conversely, DNA methylation drives the formation of heterochromatin and gene silencing. DNA methylation and histone modification pathways are therefore dependent on one another—chromatin state can direct DNA methylation which itself can equally define chromatin state—and this bidirectional cross-talk is mediated by biochemical interactions between both histone-modifying enzymes and DNA methylation enzymes in response to upstream cell signaling pathways (D’Alessio and Szyf, 2006). This has important implications for identifying mechanisms and molecular cascades involved in regulation of gene and protein expression at different stages of development or in response to pathological processes that resolve as metabolic, immune, nervous and behavioral systems.

Although necessary for survival, recurrent stress responses threaten survival of cells and organisms. Stable modifications in cell physiology involve induction of changes in gene expression programs by the activation of cell surface receptors, intracellular signaling pathways and activity-dependent transcription factors that modulate chromatin structure at responsive genes. The changes can affect DNA methylation, histone tail modifications, exchange of histone variants, or nucleosome occupancy by chromatin remodeling. Genome-wide and single cell transcriptomics have revealed how organisms respond to different stresses by regulating gene expression from chromatin structure to transcription, mRNA stability and mRNA translation (Valencia-Sanchez et al., 2006). Stable modulation of gene expression through chromatin modeling therefore has a central role in adaptation and resilience toward stress conditions.

The question then becomes what is physiologically different about individuals that successfully adapt to stressors and those that do not? The influence of early life experience and degree of parental-infant attachment on emotional development in children and adolescents has been comprehensively studied. Animal models of parental care provide both correlative and mechanistic connections between early life experience and development of cognitive and emotional responses to stress, allowing for control of genotype and environment. Increasingly, it has become appreciated that individual differences in maternal care can establish stable programming of brain region-specific gene expression through chromatin modifications and changes in DNA methylation (Figure 1), and modify phenotypic outcomes, including cognitive, social and stress-coping abilities in the offspring.

## Parental Investment, Chromatin Modifications and HPA Responses to Stress in Rodents

A common theme for many species is that the quality and stability of the early social context has profound influences on long-term emotional well-being. In mammals, both the degree of positive attachment in parent-infant bonding and level of parental investment appear to be important mediators of the infant’s cognitive and social-emotional development (Canetti et al., 1997). From an evolutionary perspective, differential parental allocation during the critical postpartum period provides newly born altricial animals with an ability to selectively hone gene expression profiles and physiological pathways associated with the development of reproductive and defensive systems to promote survival, growth and persistence in the given environment, as well as to program for sufficient parental investment in the subsequent generation (Gross, 2005; Klug and Bonsall, 2010). The relationship between early life experience and long-term health is mediated, in part, by maternal influences on the development of neuroendocrine systems that regulate the hypothalamus-pituitary-adrenal (HPA) axis and behavioral responses to stress. Accumulating evidence indicates the underlying mechanism for this developmental programming involves chromatin remodeling and changes in DNA methylation of specific genes expressed in the brain.

Despite their limitations, rodent behavioral models continue to represent the most efficient approach to elucidating the molecular and cellular mechanisms that underlie the etiopathogenesis of psychiatric disorders. Most importantly, rodent models offer access to brain tissue, which is essential for elucidating the circuit basis of these disorders (reviewed in Weaver, 2010, 2014). Observational studies in rats (and mice) have provided evidence for stable individual differences in two main forms of mother-pup interactions over the first week of lactation: licking/grooming (LG) and arched-back nursing (ABN) posture (Stern, 1997; Champagne et al., 2003). Maternal LG-ABN behavior during the first week of postnatal life is associated with stable programming of individual differences in responsiveness of the HPA axis, anxiety-like and cognitive performance and reproductive behavior in the rat (Weaver, 2011). As adults, the male offspring of high LG-ABN mothers show decreased expression of corticotrophin releasing factor (CRF), in the paraventricular nucleus of the hypothalamus (PVN), and a lower hormonal (corticosterone) response to stress by comparison to adult animals reared by low LG-ABN mothers (Liu et al., 1997; Caldji et al., 1998; Francis et al., 1999). In the rat, the maternal care appears stable across generations—the adult female offspring of high LG-ABN mothers are high LG-ABN towards their offspring and the offspring of low LG mothers are low LG-ABN towards their offspring (Francis et al., 1999). These effects, including those on the behavioral and neuroendocrine responses to stress, are reversed by cross-fostering, revealing direct maternal effects (Liu et al., 1997; Francis et al., 1999).

Interestingly, the maternal effects on stress responsivity in the offspring depend upon epigenetic programming of gene expression in the CNS. In comparison to offspring of high LG-ABN mothers, offspring of low LG-ABN mothers display life long enhanced DNA methylation and decreased acetylation of lysine 9 on histone H3 (H3K9) of the neuron-specific exon 1_7_ glucocorticoid receptor-alpha (GRα) promoter region, decreased NGFI-A transcription factor association, and decreased GRα expression in the hippocampus (Weaver et al., 2004, 2007, 2014); leading to disinhibition of CRF secretion and higher corticosterone levels in response to stress (Liu et al., 1997; Caldji et al., 1998; Francis et al., 1999). These group differences emerge over the first week of lactation, are reversed with cross-fostering, and remain stable through life and are potentially reversible in adulthood (Weaver et al., 2004). Additional *in vivo* and *in vitro* studies have provided several levels of insight into the underlying biological pathway. Maternal LG-ABN behavior during the first week of lactation stimulates production of thyroid hormones thyroxine (T4) and triiodothyronine (T3) and a subsequent increase in forebrain serotonin (5-HT) levels. Activation of the G-protein-coupled receptor, 5-HT_7_, in hippocampal neurons by serotonin initiates a signaling cascade that drives cAMP and cAMP-dependent protein kinase A (PKA) activation and NGFI-A expression. In the neonatal hippocampus, the transcription factor NGFI-A associates with the HAT CBP and the methyl-binding protein MBD2b and recruits them both to the exon 1_7_ GR promoter (Weaver et al., 2007, 2014). At the exon 1_7_ GR promoter, CBP increases acetylation on histone (H3K9ac), whereas MBD2b is associated with demethylation of the NGFI-A binding site. The remodeling of chromatin and DNA demethylation facilitates the stable binding of NGFI-A to the exon 1_7_ GR promoter, which then initiates transcription and drives GR expression and GR signaling in the neonatal hippocampus. The variation in methylation state of the exon 1_7_ GR promoter sequences remains consistent through to adulthood. In adulthood, the different levels of hippocampal GR expression is mediated by NGFI-A, which selectively binds and activates unmethylated exon 1_7_ GR promoter sequences.

These studies, among others (reviewed in Turecki and Meaney, 2016), suggest that the maternal behavior initiates a neural signaling cascade that directs activation of particular transcription factors to recruit and guide chromatin remodelers and DNA methylation enzymes to particular chromatin domains, allowing maternal behavior to affect several behavioral phenotypes in the offspring, including maternal behavior. Herein, both acquired and stable behavioral traits can be propagated across generations through epigenetic modifications to chromatin domains in a brain region- and genome sequence-specific manner. Support of this idea is evidenced by the widespread differences in hippocampal gene expression and cognitive function that has been observed in the adult offspring of high and low LG mothers (Weaver et al., 2006). For example, adult offspring of low LG mothers show increased cytosine methylation and decreased H3K9Ac of the glutamate acid decarboxylase (GAD)1 gene promoter and reduced *Gad1* mRNA expression in the hippocampus (Zhang et al., 2010). Additionally, these offspring show increased association of MECP2 to the brain-derived neurotropic factor (BDNF) gene promoter (Weaver et al., 2014), and a reduction in *Bdnf* expression, neuronal survival, synaptogenesis and synaptic plasticity in the hippocampus (Liu et al., 2000; Weaver et al., 2002; Bredy et al., 2003a). Consistent with this, these offspring exhibit deficits in hippocampal dependent tasks tests (e.g., spatial learning, object recognition; Liu et al., 2000; Bredy et al., 2003b). These maternal effects on BDNF, including cognitive ability, are reversed with peripubertal exposure to an enriched environment, revealing a potential nonpharmacological strategy to prevent the cognitive deficits associated with low levels of maternal care (Bredy et al., 2003b, 2004; Champagne et al., 2008).

In addition to maternal behavior, studies have shown persistent effects on offspring of paternal age (Smith et al., 2009, 2013), obesity (Ng et al., 2010; Fullston et al., 2013), enrichment (Mashoodh et al., 2012), and physiological/psychological stress (Franklin et al., 2010; Dietz et al., 2011; Hoyer et al., 2013; Mychasiuk et al., 2013; Rodgers et al., 2013; Gapp et al., 2014; Wu et al., 2016). These paternal effects could be disseminated via sperm (Dias and Ressler, 2014), facilitated by sperm miRNA (Rodgers et al., 2013, 2015; Gapp et al., 2014). However, studies (Dietz et al., 2011) utilizing *in vitro* fertilization following chronic paternal stress (social defeat) further support the theory that the effects of paternal stress experience on social, emotional and cognitive development in the offspring are propagated by variations in maternal behavior. The differential allocation hypothesis suggests that the dam can detect the prior experiences of potential mates through variation in his behavior and/or chemical cues, and then vary her own reproductive investment accordingly, including offspring rearing strategies. For example, dams mated with males that had been reared in an enriched environment, show increased LG-ABN behavior toward their offspring (Mashoodh et al., 2012). Consistent with this, early rearing in semi-naturalistic housing (SNH) has profound effects on offspring development—seizure severity and number of CRF-immunoreactive neurons were reduced in adolescent rats raised in SNH compared to offspring reared in standard housing (Korgan et al., 2014, 2015). Histone acetylation of the *crf* gene promoter may play a role in determining long-term sex-specific regulation of HPA endocrine function, evidenced by: (1) sex differences in *crf* gene promoter methylation and mRNA expression (Sterrenburg et al., 2011); and (2) reversal of maternal effects on stress responses by HDAC inhibitor (HDACi) treatment (Weaver et al., 2006). Consistent with this, genetic disruption of *Mecp2* in the PVN resulted in sex differences in *Crf* mRNA expression and corticosterone secretion in response to stress (Fyffe et al., 2008), although sex-differentiation mechanisms remain unclear. Taken together, these findings suggest that preconception paternal stress and housing could potentially influence the development of defensive (emotional) behaviors through differential maternal allocation to offspring.

## Chromatin Modifications and HPA Responses to Stress in Humans

Since the initial reports of epigenetic regulation of hippocampal GR expression, several studies have associated GR gene methylation status with parental stress, early-life adversity, and have attempted to determine the extent to which findings from model animals are transferable to humans (reviewed in Turecki and Meaney, 2016). Studies investigating methylation of the GR exon 1_7_ in rats or GR exon variant 1_F_ in humans in conditions of negative early-life social environments report increased GR promoter methylation within or proximal to the NGFI-A binding site. Consistent with these findings, differential methylation profiles of many genes supporting HPA function have now been shown to be environmentally regulated. For example, childhood maltreatment predicts the methylation status of the FK506 binding protein 5 (FKBP5) gene, which encodes for a functional regulator of GR protein signaling. The primary mechanism of GR signaling is as a transcription factor and the pleiotropic and organism-wide effects are strongly associated with development-related pathways. Tissue specificity is modulated by enzymatic conversion of the ligand cortisol to an inactive form, cortisone. FKBP5 decreases cortisol binding and prevents nuclear translocation of GR. Childhood maltreatment is associated with an *FKBP5* genotype-dependent demethylation of a distal enhancer, resulting in enhanced FKBP5 expression and reduced GR function (Klengel et al., 2013). Herein, in response to early social adversity, many labile genes coordinate in a tissue-specific fashion to collectively contribute to the increased HPA responsivity to stress, which may help explain the vast majority of the inter-individual variation in gene expression and social-emotional behavior in animal models of maternal care (Weaver et al., 2006).

Taken together, increased GR promoter methylation represents a general epigenetic mark of early-life stress that could potentially be a useful biomarker for human populations. Increased DNA methylation of the human *GR* gene promoter in peripheral blood lymphocytes has been associated with childhood maltreatment in individuals with borderline personality disorder, suggesting that peripheral blood could represent a proxy of the epigenetic modifications of the *GR* gene promoter occurring in the CNS (Perroud et al., 2011). Indeed, the extent of human GR gene promoter methylation shows a strong positive correlation to the reported experience of childhood maltreatment decades earlier (Perroud et al., 2014). Analysis of peripheral blood cells from adults with posttraumatic stress disorder (PTSD) revealed distinct DNA methylation and concomitant transcriptional changes in patients with a history of early abuse (Mehta et al., 2013). Together the results from these studies and others (Roberts et al., 2014) suggest the degree of DNA methylation in stress-related psychiatric disorders may have implications not only for the development of more efficient preventive and therapeutic approaches, but also in predicting and monitoring treatment.

## Chromatin Modifications and Learning and Memory and Neurodevelopmental Disorders

Learning and memory are subject to rigorous epigenetic remodeling involving multiple mechanisms of neuronal chromatin modifications in the brain to produce persistent alterations in synaptic signaling, organization, morphology and cognitive function (for review see Jarome et al., 2014; Heyward and Sweatt, 2015). Contextual fear memory formation and its initial maintenance is hippocampal dependent. Neuronal activity in the hippocampus of mice induces active DNA demethylation or *de novo* methylation (Guo et al., 2011), and targeted knockouts of DNA *de novo* methyltransferases cause learning and memory impairments (Feng et al., 2010). Memory undergoes systems consolidation over ~3 weeks, so that the remote memory becomes hippocampal independent. Similarly, retrieval of conditioned place preference memory is also dependent on DNA methylation in the prelimbic cortex (Miller et al., 2010; Day and Sweatt, 2011). Although knockout of the TET1 protein (which promotes 5hmC formation and active demethylation) results in compensatory upregulation of Tet2, Tet3 and other genes required for demethylation (e.g., Gadd45b, Smug1, Apobec1, Tdg; Jarome et al., 2015; Kumar et al., 2015), overexpression of TET1 protein (Kaas et al., 2013) and DNA methylation inhibition (Telese et al., 2015; Halder et al., 2016) result in increased expression of several neural plasticity-related genes (e.g., *Bdnf, Cobl, Reelin, PP1, Calcineurin, Vrk1*) and impaired contextual fear memory. Alterations in histone methylation (Schaefer et al., 2009) and acetylation (Guan et al., 2002) influence long-term memory formation and synaptic transmission. Accordingly, HDAC activity stimulates chromatin compaction, reducing synaptic plasticity and impairing memory formation (Guan et al., 2009). Inhibition of HDAC (by SB) can enhance memory consolidation in young (Yuan et al., 2015) and older animals (Blank et al., 2015). Histone methylation status is also critical in memory formation (Wang et al., 2015).

Disruptions to genes encoding the enzymatic proteins and metabolic intermediates that mediate DNA methylation and chromatin remodeling have profound effects on human neurobehavioral development. For example, functional polymorphisms in the gene encoding methylenetetrahydrofolate reductase (a regulatory enzyme in folate metabolism) results in altered SAM availability and are linked to the increased risk of psychiatric disorders (Miller et al., 1994; Poirier et al., 2001). On the other hand, a mutation in the gene encoding ATRX (a regulatory enzyme in chromatin remodeling) results in an X-linked form of mental retardation associated with alpha thalassaemia (ATRX syndrome; Picketts et al., 1996). The loss of ATRX function causes defective H3K9me3 binding, related to changes in the binding pocket and ability of the ATRX-DNMT3-DNMT3L (ADD) histone reader to recognize methylation patterns at specific lysine residues (Iwase et al., 2011; He et al., 2015). Further, ATRX knockouts show significant chromatin instability, even *in utero* (De La Fuente et al., 2015). This begs the question regarding the functional role of ATRX in normal cognitive and emotional development.

The two best characterized examples of the effects of epigenetic changes on cognitive function relate to dysregulation of *MeCP2* and *CBP*, which are crucial for mediating precise gene expression in neurons (Chen et al., 2003; Martinowich et al., 2003). In humans, genetic mutations in *MeCP2* and *CBP* are associated severe forms of intellectual disability—Rett syndrome and Rubinstein-Taybi syndrome (RTS), respectively—as well as increased anxiety-like behaviors (Amir et al., 1999; Alarcón et al., 2004).

The phosphorylated (active) form of MeCP2 binds broadly throughout the genome, affecting chromatin remodeling, dendritic and synaptic development and hippocampus-dependent memory (Skene et al., 2010; Li et al., 2011). Knocking out MeCP2 in inhibitory neurons causes symptom-specific effects, suggesting a substantial role for GABA-ergic dysregulation in Rett syndrome (Ito-Ishida et al., 2015). BDNF expression is also disrupted in MeCP2-deficient models. In neurons, this could be regulated by a MeCP2 mutation-induced overexpression of miR-15a, which can disrupt the BDNF pathway, and thus, neuronal maturation and dendritic morphogenesis (Gao et al., 2015). SUMOylation of MeCP2 enhances *Bdnf* mRNA, LTP and memory performance (Tai et al., 2016). Likewise, BDNF overexpression reversed many of the social, cognitive and physiological deficits observed in the *MeCP2* mutant mice (Shahbazian et al., 2002; Chang et al., 2006). Truncated MeCP2 mice show cell-type independent changes in *Bdnf* mRNA isoform expression, but MeCP2-induced effects are specific to *Bdnf* exon VI in astrocytes (Rousseaud et al., 2015). MeCP2-deficient astrocytes have significantly decreased microtubule-dependent vesicle transport and correlate to Rett-like anxiety and locomotion deficits (Delépine et al., 2016). MeCP2 is the main 5hmC-binding protein in the mammalian brain and MeCP2 bound 5hmC facilitates gene transcription (Mellén et al., 2012). However, MeCP2’s binding to 5hmC is disrupted by the Rett-causing mutation R133C. These findings provide a potential model of how 5mC, 5hmC and MeCP2 regulation of chromatin structure and gene expression may be disrupted in Rett syndrome (Bedogni et al., 2016).

RTS on the other hand, is associated with a mutation of the CBP HAT domain resulting in decreased genome-wide histone acetylation and cognitive deficits later in life (Kalkhoven et al., 2003). In humans, single exon or whole gene mutations in *cbp* flanking regions do not cause a differential diagnosis of RTS, suggesting that these flanking regions are complementary but not critical in the etiology of a clinical phenotype (Rusconi et al., 2015). Anatomically, RTS individuals show structure abnormalities related to deficits in activity dependent development and neural plasticity (Korzus et al., 2004), and display delayed myelination, neural dysgenesis, including cortical abnormalities and a thin corpus callosum and cognitive dysfunction early in life (Roelfsema and Peters, 2007; Lee et al., 2015). Mice carrying a heterozygous null mutation of *CBP* also exhibit genome-wide histone hypoacetylation show severe cognitive dysfunction early in life (Josselyn, 2005). Haploinsufficient *CBP* mice exhibit reduced activation of CBP by atypical protein kinase C (aPKC), hypoacetylation of neural genes and decreased precursor differentiation in the fetal brain as well as reduced vocalization early in postnatal life (Wang et al., 2010). Herein chromatin analysis has provided molecular insights into the critical functions of CBP that have simplified our understanding of the complex RTS pathology.

Lastly, metabolic aberrations and epigenetic regulation of gene expression in neurodegenerative disease may open the door to additional treatment options. For example, the fatal neurodegenerative disorder Niemann-Pick Type C (NPC) is caused in most cases by mutations in *NPC1*, which encodes the late endosomal NPC1 protein (Ory, 2004; Vance and Peake, 2011). Alterations in amino acid metabolism and epigenetic changes in the cerebellum have been identified in pre-symptomatic stages of NPC disease (Kennedy et al., 2016). Decreased expression of DNMt3a and MBD proteins, reduced DNA methylation in the molecular and Purkinje cell layers, demethylation of genome-wide repetitive LINE-1 elements and hypermethylation in specific promoter regions of single-copy genes in NPC1-deficient cerebellum at early stages of the disease representing previously unrecognized mechanisms of NPC pathogenesis. Deeper insight into the role of metabolic aberrations and epigenetic regulation of gene expression in NPC1-deficient brain may open the door to additional treatment options. Taken together, these studies demonstrate that dysregulation of chromatin remodeling enzymes and their modifications in chromatin structures is sufficient to cause profound deficits in neuronal plasticity and cognitive function abnormalities, underlying causes of neurodegenerative and neuropsychiatric disorders.

## Chromatin Modifications and Neuropsychiatric Disease

Stress induced changes in DNA methylation and histone modifications that fine tune HPA axis function may contribute to altered memory formation and vulnerability to mood disorders. Indeed, depression-related behavior and action of antidepressant medications have long been linked to chromatin remodeling enzymes that alter chromatin domains to regulate gene activity (reviewed in Daskalakis et al., 2015). As described above, neurotrophins such as BDNF promote the genesis, survival, development, and function of neurons important in mediating stress and depressive responses. Genetic blockade of neurogenesis (proliferating and immature neurons) in the hippocampus results in increased anxiety- and depression-like behaviors in adult animals (Snyder et al., 2011; Sakharkar et al., 2016). Mice exposed to stressors early in life show decreased CBP (which has HAT activity) and histone H3K9ac association with the BDNF IV promoter and decreased BDNF expression and neurogenesis inhibition in the hippocampus and increased anxiety- and depression-like behaviors in adulthood (Blaze et al., 2015). These results indicate that not unlike low maternal care early in life, stress exposure decreases CBP levels and histone H3 acetylation in the hippocampus, which potentially decrease BDNF expression and inhibit neurogenesis that may be involved in stress-induced behavioral abnormalities, including adult onset of mood disorders.

Repeated exposures to social defeat stress in rodents, cause a robust depression-like phenotype marked by anhedonia, anxiety and social-avoidance behaviors (Nestler and Hyman, 2010). The “chronic social defeat” model of depression (Berton et al., 2006) is a behavioral paradigm in which the animal is exposed to a more aggressive animal of the same species. When brought together again, animals chronically exposed to this stressful event tend to avoid contact with the aggressor (Tsankova et al., 2007). In mice, social avoidance results in altered chromatin and transcriptional states of many of growth factors, including BDNF (Duman and Monteggia, 2006; Castrén et al., 2007), CNTF (Kokoeva et al., 2005; Grunblatt et al., 2006), FGF (Evans et al., 2004), VGF (Thakker-Varia and Alder, 2009), VEGF (De Rossi et al., 2016), TGF (Lee and Kim, 2006), Wnt (Hiester et al., 2013), and IGF (Hoshaw et al., 2005). For example, social avoidance results in increased transcriptionally repressive H3K27me2 levels and decreased expression of hippocampal *Bdnf* splice variants (*Bdnf III and Bdnf IV*, Tsankova et al., 2006). Similarly, chronic social defeat stress was found to increase the repressive mark H3K9me3 in the hypothalamic *orexin* (*hypocretin*) gene promoter—a neuropeptide implicated in normal emotion processing (Lutter et al., 2008). Chronic administration of the widely used antidepressant imipramine increased markers of transcriptional activation H3K9/K14ac and H3K4me2 and reversed the repression of the *Bdnf* transcripts induced by defeat stress (Tsankova et al., 2006; Wilkinson et al., 2009). Other classes of antidepressants have also been shown to enhance H3K4me2 levels (Lee et al., 2006b), gene expression, cell proliferation, survival and apoptosis (Erburu et al., 2015), reverse social avoidance behavior and decrease neuroinflammatory signaling in mice, following social defeat (Ramirez et al., 2015).

The effects of imipramine on H3K9/K14ac appear to associate specifically with HDAC5 activity (Tsankova et al., 2006). Hdac5 overexpression blocks the enhanced H3K9/K14ac and *Bdnf* splice variant expression responses to the antidepressant imipramine (Tsankova et al., 2006), suggesting a potential for HDAC inhibitors in the treatment of depression. Indeed, several HDAC inhibitors, including SB (Tsankova et al., 2006), Entinostat (MS-275; Covington et al., 2009), and suberoylanilide hydroxamic acid (SAHA; Covington et al., 2009), have demonstrated antidepressant qualities and upregulate BDNF, NGF and GDNF (Valvassori et al., 2014) as well as reduce *Hdac5* expression in the hippocampus, reversing depression like phenotypes in models of chronic social defeat (Schroeder et al., 2007; Covington et al., 2015). Like social defeat stress, early life stress increases vulnerability to depression-like behavior (in rodents), which appears to be mediated through epigenetic programming at the BDNF IV promoter (Seo et al., 2015) via several histone modifiers (Pusalkar et al., 2016). Similarly, early life maltreatment is capable of decreasing H3K9/K14ac at the BDNF IV promoter (Blaze et al., 2015).

Chronic social defeat stress decreased *Hdac5* mRNA levels in the nucleus accumbens (NAc; Renthal et al., 2007), while imipramine rescues *Hdac5* mRNA expression levels in animal models of chronic social defeat (Erburu et al., 2015; Serchov et al., 2015). Accordingly, *Hdac5* KO animals showed depression-associated behavior but no effects of imipramine treatment. Additionally, *Hdac2*—but not of *Hdac1* or *Hdac3*—levels were reduced in the NAc of mice following chronic social defeat and in human *post mortem* NAc tissue from clinically depressed individuals (Covington et al., 2009). Indeed, HDAC inhibitors themselves are capable of reversing depression like phenotypes (Covington et al., 2015) and different types of HDAC inhibitors may be effective as antidepressants by each modifying distinct cellular targets. For example, in the rat, chronic antidepressant treatment with fluoxetine increases *Hdac2* mRNA expression and H3K9/K14ac levels, and enhances expression of *MeCP2* and *MBD1* in the frontal cortex and hippocampus (Cassel et al., 2006). HDAC inhibition may therefore enhance the effectiveness of antidepressants through enhancing chromatin access. For example, while fluoxetine treatment is only anxiolytic in Balb/C mice with HDAC II inhibition, combination therapy leads to enhanced acetylation association (Schmauss, 2015) and TET1-mediated DNA demethylation (Wei et al., 2015) of BDNF promoter and increased transcription. Optogenetic models of gene regulation (Konermann et al., 2013; Polstein and Gersbach, 2015) promise to provide further insight into the specific molecular mechanism(s) that underlies these effects, crucial for future drug development and treatment strategies for mood disorders.

The discovery of biomarkers and the ability to target etiological disease epigenetic changes (epimutations) in psychiatric disorders may improve their diagnosis, treatment and even their prevention. Abnormal GABAergic transmission and altered GABA-related gene methylation have been associated with SCZ, MDD and suicidal behavior in humans (Schmidt and Mirnics, 2015). For example, compared with control individuals the GABA-A α1 receptor and *Bdnf* exon IV promoter regions are hypermethylated in prefrontal cortex tissue from individuals with depression that have died by suicide (Poulter et al., 2008; Keller et al., 2010). These individuals also show increased forebrain *Dnmt3b* mRNA and protein expression. DNMT3a levels in the DG have also been shown to predict resilient/susceptible depressive phenotypes (Hammels et al., 2015). Suicidal behavior has also been associated with altered chromatin remodeling and DNA methylation and aberrant loss of transcriptional and transcription protein synthesis capacity. For example, *Ribosomal RNA* (*rRNA*) promoter methylation (Brown and Szyf, 2007, 2008) is enhanced in the hippocampus (but not the cerebellum) of individuals who died by suicide who were victims of abuse during childhood (McGowan et al., 2008). Epigenome-wide studies have identified several DNA methylation alterations in genes involved in both normal brain development and neuropsychiatric pathologies (Mill et al., 2008). Some of these epigenetic changes are sex-specific and either inherited or acquired before birth (Kaminsky et al., 2012). Interestingly, the relationship of psychosocial stress with psychiatric illness is most evident in neuroses, followed by depression and SCZ, which is influenced not only by the nature of the challenge, but also by the individual’s biological vulnerability (i.e., genetic variation) and ability to cope (i.e., resilience; Schneiderman et al., 2005). Given that neuropsychiatric disorders in adolescence and adulthood appear to have their origins in pathways that begin much earlier in life, this demonstrates the importance of preventive early intervention programs, especially those targeting early developmental antecedents such as anxiety to prevent the onset of severe mental illness (Uher et al., 2014).

## Concluding Remarks and Future Perspectives

While developmental genetic studies continue to enhance our understanding of phenotypic variation in human health and disease pathologies, emerging evidence suggests that chromatin remodeling potentially plays an important role in mediating the effects of early experience on life long programming of defensive responses to stress and stress-induced pathologies in offspring. Regulation of epigenetic programs by metabolic intermediates is emerging as an important mechanism of biological integration of distinct cellular functions. A full understanding of the link between intermediary metabolites and chromatin regulators will require the development of highly sensitive and selective sensors that measure metabolite concentrations in different organs and cellular compartments, drawing upon advances in single-cell analyses (e.g., FRET, molecular beacons, optogenetics) and genome editing technology (e.g., the CRISPR-Cas9 system). Such exploratory research in models of human complex disease (including metabolic and neurodegenerative disorders) may help to distinguish between the cause or consequence of genetic and epigenetic variation and allow a comprehensive evaluation of combinational epigenetic therapies (including HDACi and DNMTi inhibitors) on habitual functions (such as learning and memory) from a developmental perspective. Determining how epigenetic mechanisms serve as a conduit for gene-environment interactions is complex, especially when they pertain to early life programming and transmission of antecedent personality and behavioral traits and the emergence of severe mental illness. Accordingly, the nature of gene misregulation conferring risk also has broad ranging implications for our understanding of personality and the interrelations between physiology and pathology of emotions.

## Author Contributions

ICGW, ACK, KL, RVW, ASH and DG contributed equally to the preparation, writing, design, editing and proof reading this review.

## Funding

The preparation of this review was supported by a Discovery Grant from the Natural Sciences and Engineering Research Council of Canada to ICGW (Grant RGPIN-2013-436204).

## Conflict of Interest Statement

The authors declare that the research was conducted in the absence of any commercial or financial relationships that could be construed as a potential conflict of interest.
